# Chemical Composition and Nutritive Value of Sea Buckthorn Leaves

**DOI:** 10.3390/molecules29153550

**Published:** 2024-07-28

**Authors:** Paulina Bośko, Wioletta Biel, Robert Witkowicz, Ewa Piątkowska

**Affiliations:** 1 Department of Monogastric Animal Sciences, Division of Animal Nutrition and Food, West Pomeranian University of Technology in Szczecin, Klemensa Janickiego 29, 71-270 Szczecin, Poland; paulina.bosko@zohomail.eu; 2 Department of Agroecology and Crop Production, University of Agriculture in Krakow, Mickiewicza 21, 31-120 Krakow, Poland; robert.witkowicz@urk.edu.pl; 3 Department of Human Nutrition and Dietetics, Faculty of Food Technology, University of Agriculture in Krakow, Balicka 122, 30-149 Krakow, Poland

**Keywords:** *Hippophae rhamnoides* L., by-products, proximate composition, genotype, amino acids, chemical score, essential amino acids index, dietary fiber

## Abstract

Sea buckthorn leaves (SBT_LVs) form notable by-product during harvesting and post-harvest management of the berries. It is already known that sea buckthorn berries are important for their chemical composition and based on this, they occupy a wide field in nutrition. SBT_LVs also have a rich chemical composition, like the berries. The aim of this study was to describe these by-products in the context of protein and complex carbohydrates–dietary fiber fractions, including qualitative and quantitative composition of amino acids. Proximate composition, amino acids, nutritional values of the protein, and dietary fiber fractions of SBT_LVs of four cultivars (cvs.) Ascola, Habego, Hergo, and Leikora were assessed. SBT_LVs from different years of the study had statistically different levels of crude protein, ether extract, crude ash, and nitrogen-free extract (NFE), confirming that the quality of the raw material (leaves) can be significantly modified by habitat conditions. The largest fraction of dietary fiber was neutral detergent fiber (NDF), including the sum of hemicellulose, cellulose, and lignin, followed by the acid detergent fiber fraction (ADF), consisting of lignin and cellulose. The content of essential amino acids in SBT_LV protein was high. Overall, this study confirms that SBT_LVs hold promise as a valuable resource for use as a food ingredient, functional food, and dietary supplement for both humans and animals.

## 1. Introduction

The world population is rapidly increasing, so that we need to increase global food production by 70–100% in order to feed the world in 2050 (for a population of 10 billion) [1,2]. This phenomenon will have serious consequences, including an additional increase in global greenhouse gas (GHG) emissions resulting from livestock production (pork, chicken meat, beef, dairy, and eggs) [3]. Sustainable development goals (SDGs) are the only way for mankind to survive, minimize negative effects on the environment, and keep the planet’s population healthy. An increase of 50% in animal feed is necessary; thus, to save the forests, it is extremely important to study the huge amounts of agricultural by-products and use these in the best way possible for animal feed and food. Sea buckthorn (*Hippophae rhamnoides* L.), which belongs to the *Elaeagnaceae* family, is an ornamental shrub that is used for soil reclamation and improvement due to its ability to fix nitrogen through its roots [2,4]. It is a plant that contains many valuable nutrients and bioactive substances. For centuries, sea buckthorn (SBT) has been used in folk medicine in different parts of the world. In ancient Tibet, it was used for gastrointestinal disorders; in Mongolia, it was used as a sedative; the Greeks used it as a veterinary remedy [5,6,7]. In 2018, the eighth international conference dedicated entirely to sea buckthorn was held in China, indicating a clear growing interest in this plant [8,9]. Sea buckthorn fruit, pulp, and seed oil are widely used in human nutrition (in various forms, from raw to juices, jams, and oils) and are popular feed additives in livestock feeding [10,11,12]. Sea buckthorn leaf is a by-product of sea buckthorn cultivation and is usually discarded as waste. However, not only sea buckthorn berries, but also the leaves of this plant (both fresh and dried) have been recognized as a valuable source of nutrients and bioactive compounds [13,14,15]. An interesting process of change in the food industry is currently taking place in technologies related to the processing of plant by-products [16]. This includes, for example, the pomace left over from the pressing of juice and seeds [17]. Companies use broccoli leaves and stalks as additives in herbal infusions. Agro-food waste still contains high amounts of essential nutrients. Experimental results have shown that sea buckthorn leaves and twigs significantly reduce oxidative stress levels, in part due to the presence of proanthocyanidins [18]. The use of SBT_LVs in animal feed can be practiced to obtain value-added animal products and contribute to the circular economy [19,20]. In the light of the fact that food waste is a global problem, using all parts of vegetables and fruits is the most adequate sustainable solution. A sustainable bio-economy requires that by-products and side-streams in agricultural and food production are reinserted into the value cycle, a concept also called valorization or upcycling. The concept constitutes an important sustainability-oriented innovation practice contributing to waste reduction and efficient resource use. SBT_LVs are a valuable raw material, not only because of their content of bioactive compounds and their antioxidant properties but also because they can serve as a partial replacement for previously typical protein sources, especially in livestock nutrition [21]. Various parts of the sea buckthorn plant (woody verdure, seeds, leaves, barks, branches) have a high protein content compared with other berry plants’ leaves [22], but the most considerable amounts of protein (14–25 g/100 g dry matter) are found in sea buckthorn leaves, and for this reason, they are used as an unconventional source of protein in animals and human food [23,24]. Based on the results of experiments [25], SBT_LVs should be harvested from late July to early August since leaf protein content peaks around this period of time and starts to decline significantly by the middle of August. SBT_LVs are a source of protein and valuable amino acids such as lysine, methionine, and cysteine [14,26]. Thus, SBT_LVs are promising for the creation of drugs and dietary supplements [27]. In addition, one of the important health-promoting aspects of sea buckthorn leaves is their high fiber content [24]. Dietary fibers, such as mainly non-digestible carbohydrates, are one of the most heterogeneous groups of compounds that are found in natural resources, providing various health-promoting effects via regulating gut microbial composition [28]. SBT_LVs can be considered as a source of dietary fiber and other valuable nutrients contributing to health promotion. Therefore, the aim of this research was to assess the chemical composition of SBT_LVs, with particular emphasis on (1) protein and the qualitative and quantitative composition of amino acids, and (2) complex carbohydrates–dietary fiber fractions.

## 2. Results and Discussion

Sea buckthorn products are reported to be a source of nutrients and biologically active substances [29,30], but the specific cultivar and year of harvest are important factors that affect the content and composition of SBT_LVs. For these reasons, this study investigated the chemical composition of SBT_LVs from four cultivars from three harvest years.

The contents of basic nutritive components in the examined SBT_LVs are illustrated in Table 1. SBT_LVs from different years of the study had statistically different levels of crude protein, ether extract, crude ash, and nitrogen-free extract (NFE), confirming that the quality of the raw material (leaves) was significantly modified by habitat conditions. SBT_LVs are good sources of crude protein (CP) (from 15.66 to 20.77 g/100 g DM). SBT_LVs are considered to be a good source of protein, with differed significantly between the studied cultivars (Table 1). Leaves of the Hergo cv. contained 17.18 g CP/100 g DM, while in the leaves of the Habego cv., the value was 19.08 g/100 g DM (Table 1). In the study by Liang et al. [31], the CP content ranged from only 13.5 g/100 g DM in sea buckhorn branches to 17.5 g/100 g DM in leaves. Jaroszewska and Biel [24] reported as much as 24.97 g CP in 100 g DM of SBT leaves.

One special feature of sea buckthorn berry is the high oil content in the soft parts, in addition to the oil found in the seeds [32,33]. SBT_LVs are also a source of raw fat, which is well absorbed by human and animal bodies [20]. Leaves of SBT contained up to 6.87 g EE in 100 g DM. The Ascola cv. contained significantly less crude fat (as EE) than other cultivars (5.29% and 5.84–5.97% DM, respectively). The obtained results were consistent with an earlier study by Jaroszewska and Biel [24], who reported a 5.31–5.67% fat level in SBT_LVs.

The main components of dry matter in SBT_LVs are carbohydrates (CHOs)–total carbohydrates (TC) as NFE and crude fiber (CF). CHOs perform numerous essential roles in living organisms, monosaccharides being the major source of energy for human and animals metabolism, and polysaccharides serving as stores of energy and structural components [34,35]. NFE consisting of simple sugars (mono-and disaccharides), digestible starch and hydrolysis products, dextrins, and organic acids comprised the dominant share of the dry matter. The tested leaves contained on average 60% DM of NFE. Crude fiber included the sum of fibrous substances (cellulose, lignin, hemicellulose, etc.) resistant to enzymes of the digestive tract. In the studied SBT_LVs, we measured from 9.59% (Leikora cv.) to 12.14% DM (Ascola) crude fiber. The samples collected in the first year of the experiment showed significantly least CF (10.47% DM) and most CP (20.77% DM). In an earlier study, Sheikh et al. [36] found as much as 15% DM of CF.

Currently, increasing attention is being paid to nutrient components that are difficult for the monogastric gastrointestinal tract to digest, called dietary fiber. Dietary fiber (DF) has the ability to escape digestion and absorption in the small intestine, which makes it able to affect the way other nutrients are absorbed and metabolized in the gastrointestinal tract. The functionality attributed to fiber varies based on chemical and physical structure [37], and most of the time, it is hard to make a clear differentiation among attributes due to the complexity of carbohydrates found in common feedstuffs and food for humans. The physiological functions of different dietary fibers depend to a great extent on their physicochemical characteristics, one of which is solubility [38,39]. Different reports regard DF either as a functional component for normal digestive organ functioning for organisms [40,41,42] or as an antinutrient [43,44]. As a result of the use of different definitions and assay methods in research on dietary fiber, considerable problems are encountered when attempting to determine its actual level in food. For this reason, it is crucial to define not only the total level of dietary fiber in the diet, but also its fractional composition, since individual fractions are characterized by diverse action in the human body. Therefore, in our study, we determined the share of neutral detergent fiber (NDF), acid detergent fiber (ADF), acid detergent lignin (ADL), hemicellulose (HCEL), and cellulose (CEL) fractions (Table 2).

The largest fraction was NDF, which included the sum of hemicellulose, cellulose, and lignin, followed by ADF, consisting of lignin and cellulose. SBT_LVs were characterized by low ADL and HCEL levels. Leaves of the Ascola cv. were characterized by significantly higher levels of NDF fractions.

The results obtained in the present study confirmed earlier studies of the proportion of dietary fiber fraction in SBT_LVs [24,36,45]. Substances that are part of nutritive fiber show medicinal properties used for limiting obesity, diabetes, and sclerosis. Obesity is a worldwide epidemic conferring a major public health challenge, placing increased economic burden on healthcare systems, and is the fifth leading global cause of death from cardiovascular disease and cancer [46]. Dietary fiber supplementation represent a new paradigm with great potential to enhance weight management efficacy. Studies suggested that SBT_LVs ameliorate the deleterious effects of diet-induced obesity and its metabolic complications such as adiposity, dyslipidemia, inflammation, hepatic steatosis, and insulin resistance [47].

**Figure 1:**
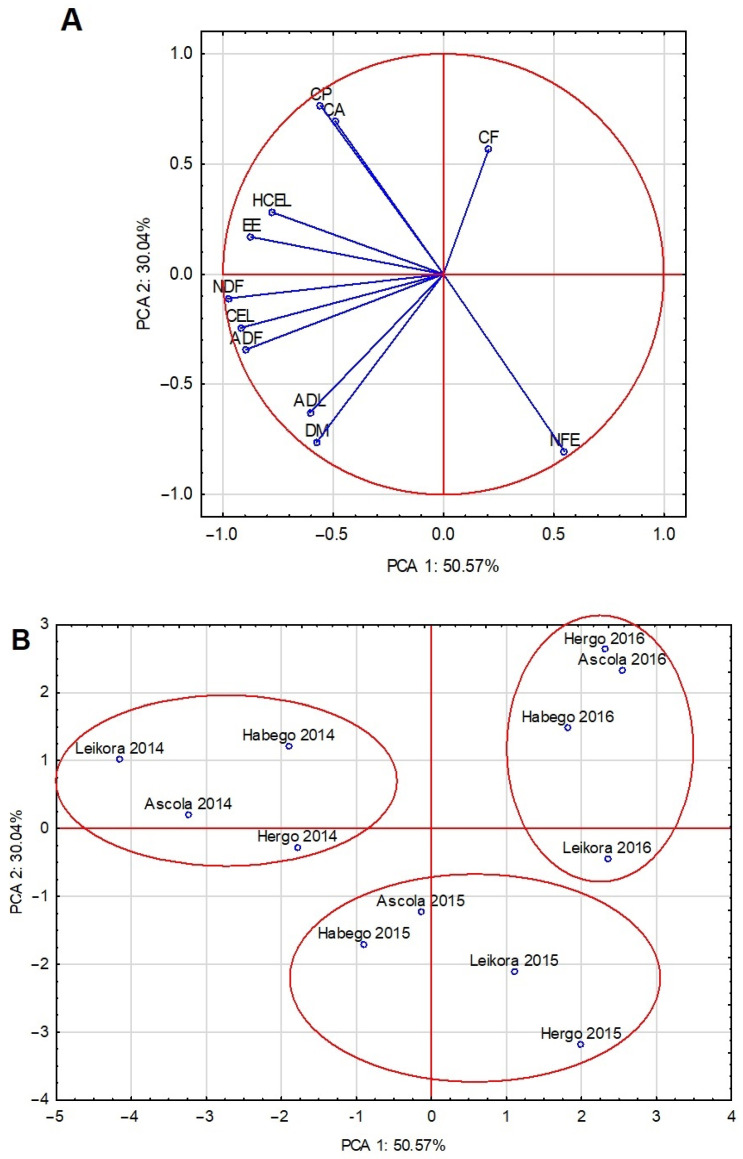
The first two principal component axes for chemical composition and fiber components in SBT_LVs for the variables (**A**) and for the scores (**B**); DM—dry matter, CP—crude protein, CF—crude fiber, EE—ether extract, CA—crude ash, NFE—nitrogen free extract, NDF—neutral detergent fiber, ADF—acid detergent fiber, ADL—acid detergent lignin, HCEL—hemicellulose, CEL—cellulose.

The PCA analysis carried out confirmed the variation in the chemical composition of the leaves of the studied sea buckthorn cultivars from different growing seasons. The climatic conditions seem to have had a dominant effect on the chemical composition of the leaves, which was confirmed by the proposed grouping shown in Figure 1A,B. This observation weakened the need for a thorough analysis of the chemical composition of the leaves of the sea buckthorn cultivars. A certain disturbance to this thesis was the location of the ‘Leikora 2016’ case in the neighborhood of the 2015 cases. The 2015 raw material was more abundant in NFE and ADL. In contrast, the 2014, especially the Leikora and Ascola cvs., was characterized by EE, HCEL, and NDF and a high positive correlation between EE and HCEL. On the other hand, in 2016, the Hergo, Ascola, and Habego cvs. contained significantly more CF.

**Figure 2:**
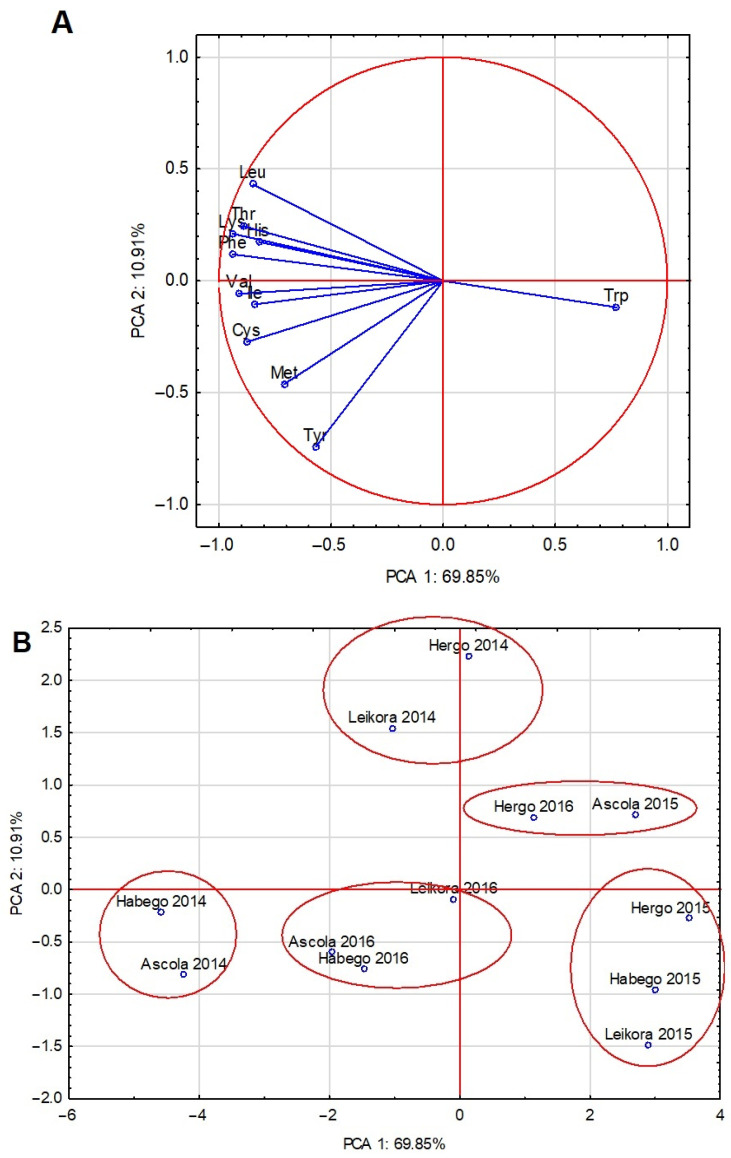
The first two principal component axes for essential amino acids in SBT_LVs for the variables (**A**) and for the scores (**B**); Lys—lysine, Met—methionine, Cys—cysteine, Thr—threonine, Ile—isoleucine, Trp— tryptophan, Val—valine, Leu—leucine, His—histidine, Phe—phenylalanine, Tyr—tyrosine.

Proteins are made up of 20 amino acids. There are few studies in the literature on the amino acid content and quality of SBT_LVs. Table 3, Table 4 and Table 5 and Figure 2 show the amino acid profile in the examined SBT_LVs and the protein nutritive value. Nutritionally, amino acids are divided into three groups—essential, non-essential, and semi-essential. Out of 20 amino acids, a human being or an animal can synthesize only some of these (NEAAs, non-essential amino acids). Semi-essential amino acids (SEAAs) are synthesized by the body but are designated essential during periods of stress. Nine amino acids, including histidine, isoleucine, leucine, lysine, methionine, phenylalanine, threonine, tryptophan, and valine, are classified as essential amino acids (EAAs) because they cannot be synthesized by human or other mammalian cells [48,49]. Arginine is regarded as an EAA in birds and fish. The content of essential amino acids in the protein of the SBT_LVs was high. In most cases, there was no significant difference between the cultivar tested in the levels of both EAAs and NEAAs. (Table 3 and Table 4). Average EAAs in the protein of SBT leaves in our own experiments amounted to 80.67 g/16 g N for a mature human (MH) and 66.96 g/16 g N for animals (without His) (Table 5). In comparison to the standards for humans and animals (MH and WE), Trp turned out to be the first amino acid (CS) limiting the quality of SBT_LV proteins in all examined samples (Table 3 and Table 5). A high content of essential amino acids (EAAs) was reflected in EAAI results, which ranged in relation to the human standard from 73 to 81% and to the animal standard from 58 to 67% (Table 5). The PCA analysis carried out on the amino acid composition of the raw material no longer allowed the cases to be grouped mainly on the basis of the growing season as clearly as the previous PCA analysis (for the basic composition). This was particularly evident in the arrangement of the 2014 cases (individual cultivars). The raw material obtained from the Habego and Ascola cultivars was clearly characterized by a higher amino acid composition (Figure 2A,B). The leaves of these cultivars were characterized by a higher content of Val, Ile, Cys, Met, and Tyr in comparison to the leaves of the Hergo and Leikora cultivars. Other observations from this analysis included the higher tryptophan content in the leaves from 2015 and the clear negative correlation of this amino acid with Thr, Lys, His, and Phe.

## 3. Materials and Methods

### 3.1. Materials

The leaves of sea buckthorn (*Hippophae rhamnoides* L.) of four cultivars (cvs.) Ascola, Habego, Hergo, and Leikora were collected from an experiment conducted at the Agricultural Experimental Station in Lipnik belonging to the West Pomeranian University of Technology in Szczecin, Poland (53° 42′ N, 14° 97′ S), in 2014–2016. The leaves for analysis were taken at full maturity of berries (which was the period from the 1st to the 15th of August). Leaves were weighed and dried at room temperature (18–22 °C) for 3–4 days. Samples were ground to 0.1 mm by use of a KNIFETEC 1095 laboratory mill (Foss Tecator, Höganäs, Sweden).

### 3.2. Chemical Analyses

#### 3.2.1. Proximate Composition

The chemical composition was determined according to the Association of Official Analytical Chemists [50]. Samples were dried at 105 °C to a constant weight to determine dry matter (method 945.15). Crude protein (N × 6.25, CP) (method 945.18) was analyzed by the Kjeldahl method, using a Büchi Scrubber B414 unit and a Büchi 324 distillation set (Büchi Labortechnik AG, Flawil, Switzerland). Crude fat (as an ether extract, EE) was analyzed by the Soxhlet extraction method with diethyl ether (method 2003.06). Crude fiber (CF) (method 962.09) was determined using an ANKOM220 Fiber Analyzer (ANKOM Technology, New York, NY, USA). Crude ash (CA) (method 920.153) was measured by burning in a muffle furnace at 580 °C for 8 h. Nitrogen-free extract (NFE) was estimated according to the following calculation [51]:

NFE (%) = 100 − % (moisture + crude protein + crude fat + crude ash + crude fibre)(1)

All determinations were expressed on a dry matter basis.

#### 3.2.2. Amino Acids

Amino acids were determined using an AAA 400 automatic amino acid analyzer (INGOS, Prague, Czech Republic). Samples were subjected to acid hydrolysis in the presence of 6 M HCl at 105 C for 24 h. Sulphur-containing amino acids were determined separately in 6 M HCl after oxidative hydrolysis (formic acid þ hydrogen peroxide, 9:1 *v*/*v*, 20 h at 4 °C). Tryptophan was determined according to the method described in AOAC [52]. Amino acid determinations were expressed on a g/16 g N basis, equivalent to g/100 g of protein.

#### 3.2.3. Estimation of Nutritive Values of SBT Protein

Chemical score (CS) was calculated on the basis of a procedure described by Block and Mitchell [53] based on comparison of the concentration ratio of the amino acid having the shortest supply (ai) (received amino acid) to that of this amino acid in a standard (as) (CS = (ai/as)/100). Two standards were used: amino acids of the food protein composition appropriate for a mature human (MH) [54,55] and amino acid composition of whole egg protein (WE) [56], considered a complete and balanced food and fodder protein.

Recommended levels of essential amino acid were as follows: Lys—5.5 and 7.0 g/16 g N, Met + Cys—3.5 and 5.7 g/16 g N, Thr—4.0 and 4.7 g/16 g N, Ile—4.0 and 5.4 g/16 g N, Trp—1.0 and 1.7 g/16 g N, Val—5.0 and 6.6 g/16 g N, Leu—7.0 and 8.6 g/16 g N, His—0 and 2.2 g/16 g N, and Phe + Tyr—6.0 and 9.3 g/16 g N, respectively, for mature human and egg protein standards [54,55,56].

Essential amino acids (EAAs) were estimated in accordance with Oser [57] in terms of the geometric mean of all the concentrations of participating essential amino acids compared with the concentration of the corresponding standard (in g/16 g N):

(2) $$\mathrm{EAA}=\sqrt[{n}]{a_{1}/a_{1s}\times 100\times \ldots \times a_{n}/a_{\mathrm{ns}}\times 100}$$

where *n* is the number of participating amino acids and ns is the number of corresponding amino acids in a standard.

In the classical Oser method [57,58], concentrations of Lys, the sum of Met + Cys, Thr, Ile, Trp, Val, Leu, His, and Phe +Tyr were considered, whereas the standard for a mature human (MH) excluded histidine.

The essential amino acid index (EAAI) was calculated as follows:

EAAI = 10 ^log EAA^ (3)

where log EAA is described by:


(4) $$\log\mathrm{EAA}=1/10(\log(a_{1}/a_{1s})\times 100+\log(a_{2}/a_{2s})\times 100+\ldots +\log(a_{n}/a_{ns})\times 100)$$

#### 3.2.4. Dietary Fiber Fractions

The fiber components were determined using the detergent method according to Van Soest et al. [59], performed with the ANKOM 220 fiber analyzer. Determination of neutral detergent fiber (NDF) was conducted on an ash-free basis and included sodium dodecyl sulphate (Merc 822050). Determination of acid detergent fiber (ADF) included hexadecyltrimethylammonium bromide (Merc 102342), while acid detergent lignin (ADL) was determined by hydrolysis of ADF samples in 72% sulfuric acid. The content of cell wall structural carbohydrates hemicellulose and cellulose was calculated as the following differences:

(5) cellulose (CEL)=ADF−ADLhemicellulose (HCEL)=NDF−ADF

### 3.3. Statistical Analysis

A two-factorial analysis of variance (ANOVA) and principal component analysis (PCA) were carried out using the STATISTICA v13.30 software (TIBCO Software Inc. [60], Palo Alto, CA, USA). Tukey’s honestly significant difference (HSD) at *p* = 0.05 was used to find the differences between means.

## 4. Conclusions

The findings clearly indicate that sea buckthorn leaves hold promise as a valuable resource for use as food ingredient, functional food, and dietary supplement for both humans and animals. What is more, SBT_LVs are not only a source of active substances, as shown in the literature, but our studies have shown them to be a valuable component in animal and human diets due to their high-quality protein with a good essential amino acid composition, making the product a partial protein substitute. Leaves, until recently treated as exclusively an agro-weed biomass, can be an important component in the human diet because of their interesting dietary fiber composition.

At a time when sustainability is an important element of a properly conducted agricultural policy, the use of this raw material is extremely valuable. It is important to emphasize the positive effect of dietary fiber on the regulation of the cerebral–intestinal axis and, consequently, on the regulation of many systems in the living organism.

## Figures and Tables

**Table 1:** Chemical composition of SBT ^1^ leaves.

Factor	Factor Level	DM ^2^	CP	EE	CF	CA	NFE
		g/100 g	g/100 g DM				
Year	2014	95.14 b ^3^	20.77 c	6.87 c	10.47 a	5.76 c	56.14 a
	2015	95.35 c	15.66 a	5.35 b	10.50 a	4.06 a	64.43 c
	2016	92.98 a	18.34 b	5.00 a	11.90 b	5.21 b	59.55 b
Cultivar	Ascola	94.22 a	18.76 c	5.29 a	12.14 c	5.19 b	58.61 a
	Hergo	94.22 a	17.18 a	5.84 b	10.21 b	4.69 a	61.49 b
	Habego	94.65 b	19.08 c	5.97 b	11.88 c	5.09 b	57.97 a
	Leikora	94.86 c	18.00 b	5.84 b	9.59 a	5.07 b	61.49 b

^1^ SBT—sea buckthorn; ^2^ DM—dry matter, CP—crude protein, CF—crude fiber, EE—ether extract, CA—crude ash, NFE—nitrogen free extract; ^3^ means with at least one same letter (a, b, c) did not differ statistically at *p* = 0.05 (for all columns and factors separately).

**Table 2:** Dietary fiber fractions of SBT ^1^ leaves (g/100 g DM ^2^).

Factor	Factor Level	NDF ^3^	ADF	ADL	HCEL	CEL
Year	2014	31.61 c ^4^	20.81 c	6.18 b	10.80 b	14.64 c
	2015	25.61 b	18.63 b	6.55 c	6.99 a	12.08 b
	2016	21.06 a	13.79 a	5.09 a	7.28 a	8.70 a
Cultivar	Ascola	27.37 c	18.90 b	6.02 a	8.47 a	12.88 b
	Hergo	24.38 a	15.92 a	5.75 a	8.47 a	10.17 a
	Habego	25.97 b	18.47 b	6.08 a	7.50 a	12.38 b
	Leikora	26.67 b c	17.68 b	5.90 a	8.98 a	11.78 b

^1^ SBT—sea buckthorn; ^2^ DM—dry matter, ^3^ NDF—neutral detergent fiber, ADF—acid detergent fiber, ADL—acid detergent lignin, HCEL—hemicellulose, CEL—cellulose; ^4^ means with at least one same letter (a, b, c) did not differ statistically at *p* = 0.05 (for all columns and factors separately).

**Table 3:** Essential amino acids (g/16 g N) of SBT ^1^ leaves.

Factor	Factor Level	Lysine	Sulfur-Containing Amino Acids		Threonine	Isoleucine	Tryptophan	Valine	Leucine	Histidine	Aromatic Amino Acid	
			Methionine	Cystine							Phenyloalanine	Tyrosine
Year	2014	5.24 b ^2^	7.93 a	1.22 b	4.49 b	3.62 b	0.27 a	4.37 b	7.19 c	2.02 c	4.18 b	3.00 a
	2015	3.90 a	7.62 a	0.92 a	3.07 a	2.83 a	0.42 c	3.66 a	4.81 a	1.17 a	3.34 a	2.78 a
	2016	4.88 b	8.07 a	1.23 b	3.97 b	3.14 a b	0.37 b	4.15 a b	6.18 b	1.93 a b	3.86 b	2.93 a
Cultivar	Ascola	4.70 a	7.86 a	1.19 a b	3.93 a	3.45 a	0.31 a	4.22 a	6.18 a	2.09 a	3.91 a	3.30 b
	Hergo	4.51 a	7.69 a	0.91 a	3.73 a	2.81 a	0.45 b	3.99 a	5.91 a	1.82 a	3.76 a	2.25 a
	Habego	4.82 a	8.25 a	1.28 b	4.16 a	3.24 a	0.33 a	4.04 a	6.60 a	1.88 a	3.82 a	3.26 b
	Leikora	4.66 a	7.70 a	1.10 a b	3.55 a	3.28 a	0.31 a	3.99 a	5.55 a	1.82 a	3.68 a	2.80 a b

^1^ SBT—sea buckthorn; ^2^ means with at least one same letter (a, b, c) did not differ statistically at *p* = 0.05 (for all columns and factors separately).

**Table 4:** Non-essential amino acids (g/16 g N) of SBT ^1^ leaves.

Factor	Factor Level	Aspartic Acid	Serine	Glutamic Acid	Proline	Glycine	Alanine	Arginine
Year	2014	10.55 c ^2^	4.12 c	8.87 a	4.79 c	5.31 c	2.87 a	4.26 a
	2015	6.19 a	2.88 a	8.88 a	3.22 a	3.52 a	2.91 a	4.65 a
	2016	8.39 b	3.50 b	9.00 a	4.00 b	4.43 b	2.93 a	4.45 a
Cultivar	Ascola	8.35 a	3.37 a b	9.30 a	3.51 a	4.41 a b	2.79 a	4.37 a
	Hergo	8.07 a	3.73 b c	8.63 a	4.34 b	4.42 a b	3.02 a	5.01 a
	Habego	9.02 b	4.01 c	8.46 a	4.03 a b	4.78 b	3.07 a	4.31 a
	Leikora	8.08 a	2.89 a	9.29 a	4.14 a b	4.06 a	2.73 a	4.13 a

^1^ SBT—sea buckthorn; ^2^ means with at least one same letter (a, b, c) did not differ statistically at *p* = 0.05 (for all columns and factors separately).

**Table 5:** Nutritional values of protein of SBT ^1^ leaves.

Factor	Factor Level	EAAI ^2^ MH ^5^	EAAI WE ^6^	CS ^3^ MH	CS WE	EAAs ^4^ MH	EAAs WE
Year	2014	41.51 b ^7^	43.52 b	26.87 a	15.81 a	80.67 b	66.96 b
	2015	33.35 a	35.11 a	42.02 c	24.72 c	72.45 a	57.94 a
	2016	38.77 b	40.70 b	34.43 b	20.82 b	80.11 b	64.75 b
Cultivar	Ascola	39.05 a	41.14 a	30.79 a	18.11 a	78.37 a	64.90 a
	Hergo	36.03 a	37.84 a	42.70 b	25.87 b	77.86 a	62.27 a
	Habego	39.80 a	41.68 a	33.17 a	19.51 a	78.85 a	64.71 a
	Leikora	36.63 a	38.45 a	31.12 a	18.30 a	75.91 a	60.99 a

^1^ SBT—sea buckthorn; ^2^ EAAI, essential amino acid index; ^3^ CS, chemical score of restrictive amino acid(s); ^4^ EAAs—essential amino acids; ^5^ ME—mature human protein standards; ^6^ WE—whole egg protein standards; ^7^ means with at least one same letter (a, b, c) did not differ statistically at *p* = 0.05 (for all columns and factors separately).

## Data Availability

All data generated or analysed during this study are included in this published article.
